# Association between pre-operative computed tomography-based adipose tissue quantification and post-transplant dyslipidemia in renal transplantation recipients

**DOI:** 10.3389/fnut.2026.1729426

**Published:** 2026-03-31

**Authors:** Yang Feng, Ming Liu, Yuechen Shi, Yuqing Yi, Xiaomeng Xu, Kexin Ma, Ke Wang, Ying Wang

**Affiliations:** 1 Department of Urology, The Second Hospital of Dalian Medical University, Dalian, Liaoning, China; 2 Department of Clinical Nutrition, Shenzhen Integrated Traditional Chinese and Western Medicine Hospital, Shenzhen, Guangdong, China; 3 School of Nursing, Jilin University, Changchun, China; 4 Department of Hepatobiliary Surgery, The Second Hospital of Dalian Medical University, Dalian, Liaoning, China

**Keywords:** body composition, dyslipidemia, intermuscular adipose tissue, kidney transplantation, visceral adipose tissue

## Abstract

**Background:**

There is an established correlation between obesity and dyslipidemia in individuals who have undergone kidney transplantation (KT). Body composition is a more accurate indicator of obesity than body mass index (BMI). However, the relationship between pre-operative body composition and post-transplant dyslipidemia remains unexplored.

**Methods:**

We analyzed 333 participants who underwent KT between 2021 and 2024. Using pre-transplantation computed tomography (CT), we assessed the cross-sectional areas of skeletal muscle (SM) and adipose tissue at the level of the third lumbar vertebra. Participants were categorized into high and low body composition groups based on the highest quartile, and into dyslipidemia and non-dyslipidemia groups according to post-transplant blood lipid levels. Skeletal muscle (SM) and adipose tissue metrics were subsequently used to model dyslipidemia after KT. Generalized estimating equations (GEE) were then employed to assess the impact of body composition on blood lipid levels at 45 days, and at 3, 6 months, and 1 year post-transplant.

**Results:**

Univariate analysis revealed that BMI (*P* = 0.006), visceral adipose tissue (VAT) area (*P* < 0.001), intermuscular adipose tissue (IMAT; *P* = 0.005), subcutaneous adipose tissue (SAT; *P* = 0.003), and skeletal muscle (*P* = 0.019) were identified as risk factors for dyslipidemia 1 year post-transplant. A predictive model was further developed, indicating that VAT was a more reliable predictor than BMI and other body composition metrics. Statistically significant associations were observed for both VAT (OR = 1.005, 95% CI: 1.001–1.009; *P* = 0.014) and VATI (OR = 1.013, 95% CI: 1.002–1.025; *P* = 0.027). Sensitivity analysis indicated that the observed effect sizes were near the detection limit for a study of this sample size, reinforcing the modest magnitude of these associations. REE analysis demonstrated that VAT influenced triglyceride (TG) levels (*P* = 0.006), high-density lipoprotein cholesterol (HDL-C) levels (*P* = 0.001), and low-density lipoprotein cholesterol (LDL-C) levels (*P* = 0.033).

**Conclusions:**

High pre-transplant VAT was associated with low HDL-C, elevated TG, and LDL-C levels after KT. These findings may have significant implications for reducing the incidence of dyslipidemia post-KT.

## 1 Introduction

Cardiovascular disease (CVD) is the leading cause of organ failure and mortality among kidney transplant (KT) recipients (1, 2). Dyslipidemia is a key pathogenic factor in the development of CVD (3). Dyslipidemia is abnormal lipid metabolism characterized by any one of hypercholesterolemia, high-density lipoprotein (HDL)-hypocholesterolemia, low-density lipoprotein (LDL)-hypercholesterolemia, and hypertriglyceridemia (3). Despite substantial evidence indicating that dyslipidemia management is crucial for the prevention of CVD (3–5), its incidence remains high following kidney transplantation (6). Furthermore, the mortality rate attributable to CVD among KT recipients is estimated to be as high as 42% (7). This phenomenon is closely linked to the use of immunosuppressive drugs, which, however, remains an unavoidable aspect of post-KT care. Therefore, identifying modifiable risk factors is essential for preventing dyslipidemia after KT.

Obesity is a principal risk factor for dyslipidemia (8). Although body mass index (BMI) is the most widely used indicator for diagnosing obesity in patients (9), it fails to accurately reflect the true physical manifestation of obesity. The role of body composition as a key risk factor for dyslipidemia has garnered significant attention in recent years. One review examined the association between body composition, BMI, and cardiovascular risk, concluding that body composition may be a stronger predictor of cardiovascular risk than BMI (9). Another large cohort study demonstrated that various adipose measures were associated with the incidence of cardiovascular diseases independently of BMI (10). A recent study found that visceral adipose tissue (VAT) levels were significantly correlated with all laboratory lipid values in a normative population (11). However, it should be noted that the current study did not examine other types of adipose tissue. For example, intermuscular adipose tissue (IMAT) serves as a reservoir for ectopic fat that accumulates under the fascia and within the muscles. Despite growing evidence of the role of body composition in dyslipidemia (11–13), few studies have explored the relationship between body composition and dyslipidemia following KT.

Computed tomography (CT) is an advanced and accurate method for evaluating body composition, regarded as the gold standard in numerous studies (14, 15). In patients who have undergone liver transplantation, pre-transplant CT serves as a valuable tool for predicting post-operative mortality (16, 17). Moreover, CT is used to measure the area of the psoas muscles (18). However, this technique has not been widely applied to assess body composition in KT recipients, and the relationship between body composition and post-transplant outcomes remains poorly understood.

**Figure 1:**
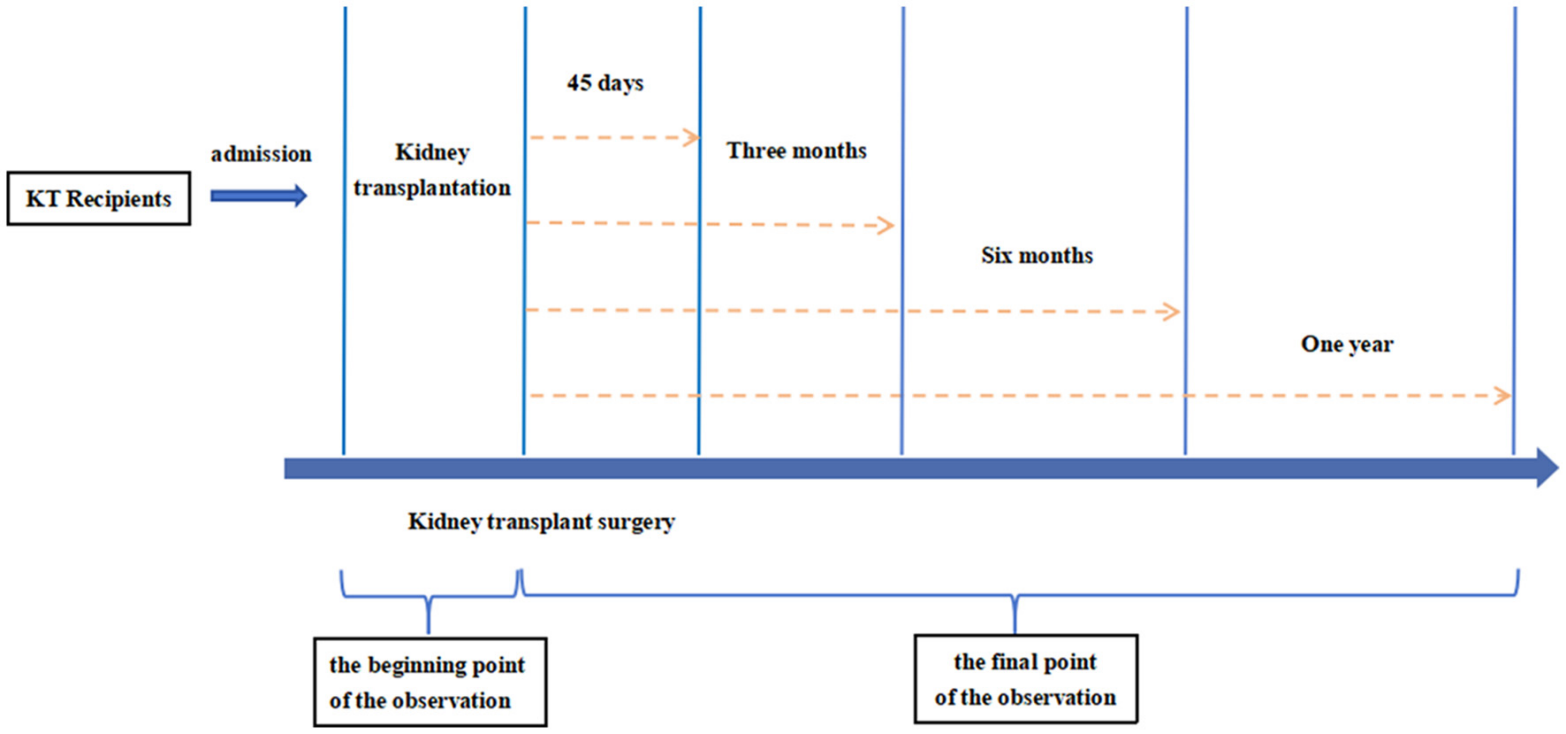
Overview of the research process. KT, kidney transplantation.

Therefore, this study aims to explore the impact of pre-transplant CT-derived body composition, including skeletal muscle (SM), SAT, VAT, and IMAT, on dyslipidemia after KT, as evidenced by the presence of any of the following: hypercholesterolemia, HDL-hypocholesterolemia, LDL-hypercholesterolemia, and hypertriglyceridemia (3). Additionally, lipid levels were measured at 45 days, 3 months, 6 months, and 1 year post-KT to further investigate the relationship between pre-operative body composition and long-term lipid levels. The study timeline is shown in Figure 1.

## 2 Methods

### 2.1 Participant selection

This study included participants who had undergone kidney transplantation (KT) and were admitted to the Second Affiliated Hospital of Dalian Medical University between June 2021 and September 2024. Participants from June 2021 to December 2022 gave verbal informed consent, while those from January 2023 to September 2024 provided written informed consent. Participants were eligible if they: (a) were aged ≥18 years; (b) were first-time KT recipients; and (c) had pre-operative abdominal CT imaging data available. Participants were excluded if they: (a) were actively taking medication for dyslipidemia prior to KT; (b) had undergone a second transplantation or combined transplantation; (c) experienced rejection after transplantation; or (d) had transplant failure. This study was approved by the Institutional Ethics Committee of the Second Affiliated Hospital of Dalian Medical University (Dalian, China; Approval No. [203]165). This research adhered to the ethical standards outlined in the Declaration of Helsinki.

### 2.2 Clinical data collection

Pre-operative data, including abdominal CT imaging, medical history, laboratory tests, and prescription information, were retrieved from the hospital's medical record system. Post-operative lipid levels, including total cholesterol (TC), triglycerides (TG), low-density lipoprotein cholesterol (LDL-C), and high-density lipoprotein cholesterol (HDL-C), were retrieved from outpatient medical records at 45 days, 3 months, 6 months, and 1 year post-KT.

### 2.3 Diagnosis of dyslipidemia after KT

According to the Chinese Clinical Practice Guidelines for Dyslipidemia in Kidney Transplant Recipients, participants who met at least one of the diagnostic criteria for dyslipidemia after KT (TC ≥ 6.2 mmol/L; LDL-C ≥ 4.1 mmol/L; HDL-C < 1.0 mmol/L; TG ≥ 2.3 mmol/L) (19) were categorized as the dyslipidemia group. Participants who did not meet the criteria were categorized as the non-dyslipidemia group.

### 2.4 Anthropometric measurement

**Figure 2:**
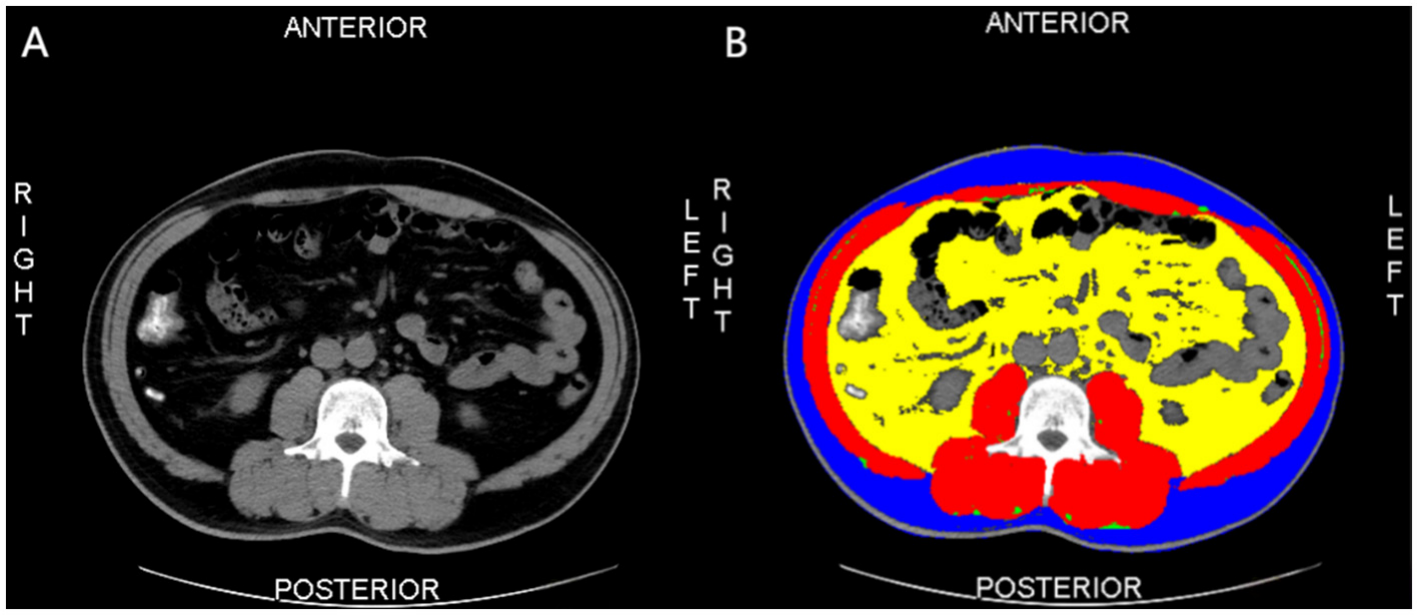
The morphometric measurement of one of the participants with dyslipidemia. This image was taken from a male, post-transplant diabetic, participant, with a BMl of 30.24 kg/m^2^, visceral adipose tissue area of 279.6 cm^2^, intermuscular adipose tissue area of 4.3 cm^2^, skeletal muscle area of 192.1 cm^2^, and subcutaneous adipose tissue area of 148.1 cm^2^. Blue: the subcutaneous adipose tissue area. Red: the skeletal muscle area. Yellow: the visceral adipose tissue area. Green: the intermuscular adipose tissue area.

A 64-slice spiral CT scanner from the Brilliance or Ingenuity series (Philips Healthcare, The Netherlands) was used to obtain abdominal CT images. Based on abdominal CT images, two consecutive images at the L3 lumbar level were selected by the investigator. Image segmentation was then performed by a trained researcher using Slice-O-matric software version 5.0 (Tomovision, Montreal, Canada) based on the anatomical structure of human muscle fat, and reviewed by an experienced radiology radiologist. A Hounsfield unit threshold of −190 to −30 was identified as IMAT; a Hounsfield unit threshold of −29 to 150 was identified as SM; a Hounsfield unit threshold of −150 to −50 was identified as VAT; a Hounsfield unit threshold of −190 to −30 was identified as SAT (20). Finally, according to the different anatomical positions of the SM, IMAT, VAT, and SAT, different tissues of the CT images were segmented. After calculating the cross-sectional area (cm^2^) of the different tissues in each image, the average of the two images was taken (Figure 2).

### 2.5 Grouping of body composition

The participants were divided into high and low groups based on the VAT, SAT, IMAT, and SM quartiles. The upper quartile values for each measure were considered high, while values in the lower and middle quartiles were considered low.

### 2.6 Immunosuppressive regimen

All participants received tacrolimus, prednisone (or methylprednisolone), and mycophenolate mofetil (MMF) therapy both before and after KT. Tacrolimus was administered orally at a dose of 0.5–3.5 mg twice daily prior to KT. Prednisone was administered orally once daily, starting with an initial dose of 5.0–20.0 mg. Prednisone may be substituted with methylprednisolone, dosed at 4 mg twice daily or 16 mg once daily. Participants' physical condition was monitored to adjust the medication dosage accordingly. MMF was administered at a dose of 750 mg twice daily prior to KT. None of the participants received anti-lipid medications during hospitalization.

The pharmaceutical regimen must be adjusted post-KT. According to existing clinical guidelines (21), while a standardized protocol for tacrolimus blood concentration testing exists, the actual frequency is often determined by surgeons based on individual patient conditions. The frequency of tacrolimus blood concentration testing post-renal transplantation is as follows: weekly for the first 3 months, biweekly for 3–6 months, every 4 weeks for 6–12 months, and every 6–8 weeks thereafter. Clinicians adjust tacrolimus dosage based on blood concentration measurements to maintain it within a specific therapeutic range. The guideline recommends monitoring tacrolimus blood levels using the 12-h trough concentration. For renal transplant patients, the target trough concentration reference values for tacrolimus are as follows: 8–12 ng/ml within 1 month post-transplantation, 6–10 ng/ml for 1–3 months, 5–10 ng/ml for 4–12 months, and 5–8 ng/ml beyond 1 year post-surgery. Additionally, abnormal tacrolimus blood levels may be influenced by various factors, including diet, medication adherence, coadministration, blood volume, monitoring methods, and individual patient differences. Therefore, clinicians at this study center made every effort to maintain patients' tacrolimus trough concentrations within the guideline-recommended target range, considering individualized treatment factors.

### 2.7 Statistical analysis

SPSS 26.0 (IBM Corp., Armonk, NY, USA) software and R (version 4.2.2; R Foundation for Statistical Computing, Vienna, Austria) were used to analyze the collected data. No more than 15% of values were missing in this study of pre-operative data (Supplementary Figure S1); however, to eliminate any bias resulting from the missing data, we filled in the gaps with multiple imputations before data analysis. There was no statistical difference between the demographic characteristics of the participants who were lost to follow-up and the study population (Supplementary Table S1). Laboratory lipid values were compared between the low and high body composition groups. Body composition and other factors were compared between the dyslipidaemic and non-dyslipidaemic groups. The normal distribution of continuous variables was described by mean ± standard deviation ($\bar{x}\pm$S). The skewed distribution of continuous variables was described by the median and interquartile range. Categorical variables are expressed as percentages (%). Categorical data were compared using the *t*-test, and Fisher's exact test was used when the data size was less than 5. The normal distribution of continuous variables was compared using the independent *t*-test. The Mann–Whitney *U* test was used for skewed distributions of continuous variables. Then, variables with *P* < 0.1 in univariate analysis were assessed for multicollinearity. To mitigate multicollinearity among correlated body composition measures (e.g., height, weight, BMI, VAT area, VATI), they were grouped into four distinct predefined sets for multivariate modeling. A backward stepwise logistic regression was used to build each model for predicting post-KT dyslipidemia. The final model was selected by comparing the AUCs of the four models. Multicollinearity diagnostics confirmed its absence in each final model (Supplementary Table S3).

A sensitivity analysis was performed to assess the study's capability to detect the observed weak associations. For key exposures (e.g., VAT, VATI), we calculated the minimum detectable odds ratio (OR) with 80% power at α = 0.05, based on the observed variance and sample size, using a standard logistic regression sample size framework.

A generalized estimating equation with an autoregressive order-1 [AR (1)] was employed to examine the variations in lipid levels between the high and low VAT, IMAT, SAT, and SM groups at different times, as the longitudinal lipid data from this study did not follow a normal distribution. A significance level of *P* < 0.05 was used to define statistical significance.

## 3 Results

**Figure 3:**
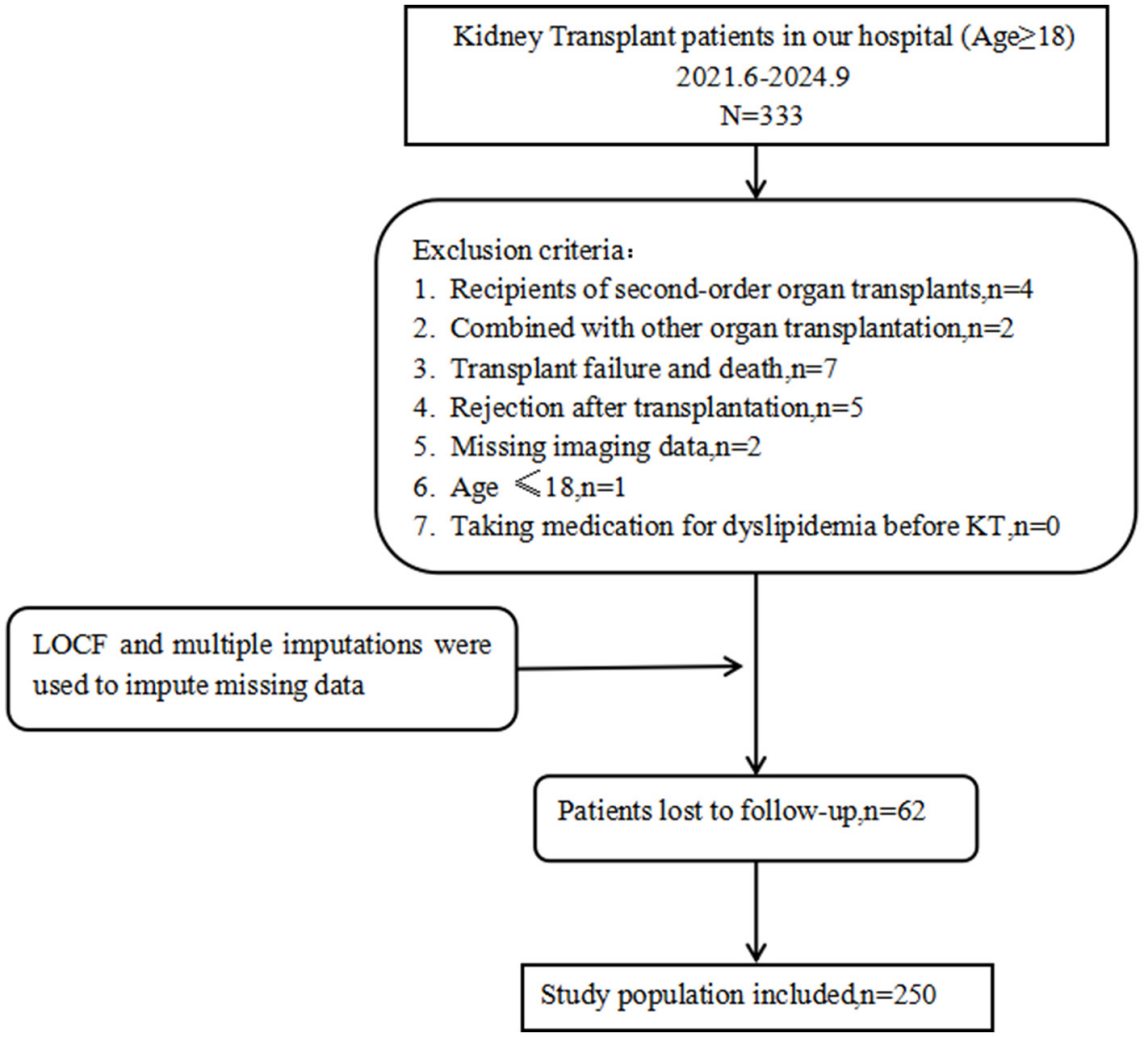
Flow diagram for the selection of the participants.

Following screening, a total of 250 participants were included in the study. Lipid analysis of the four deceased recipients revealed that all exhibited dyslipidemia prior to death. Pre-operative lipid analysis of recipients undergoing a second KT revealed that all exhibited dyslipidemia prior to surgery. In our analysis of the causes of 1-year readmissions following kidney transplantation, cardiovascular disease ranked as the fourth most common cause, after infection, acute rejection, and surgical complications (Table 1). A flowchart of participant enrollment is presented in Figure 3.

**Table 1:** Percentage of complications after kidney transplantation within 1 year.

Diseases	Percentage
Lung inflammation	(31) 12.4%
Infection of the urinary tract	(23) 9.2%
Pyelonephritis	(15) 6.0%
Cardiovascular disease	(12) 4.8%
Kidney hydronephrosis	(7) 2.8%
Drug-induced renal impairment	(7) 2.8%
Giant cell infection	(3) 1.2%
Thyroid nodule	(3) 1.2%
Gastrointestinal bleeding	(3) 1.2%

### 3.1 Epidemiology characteristics of participants

**Figure 4:**
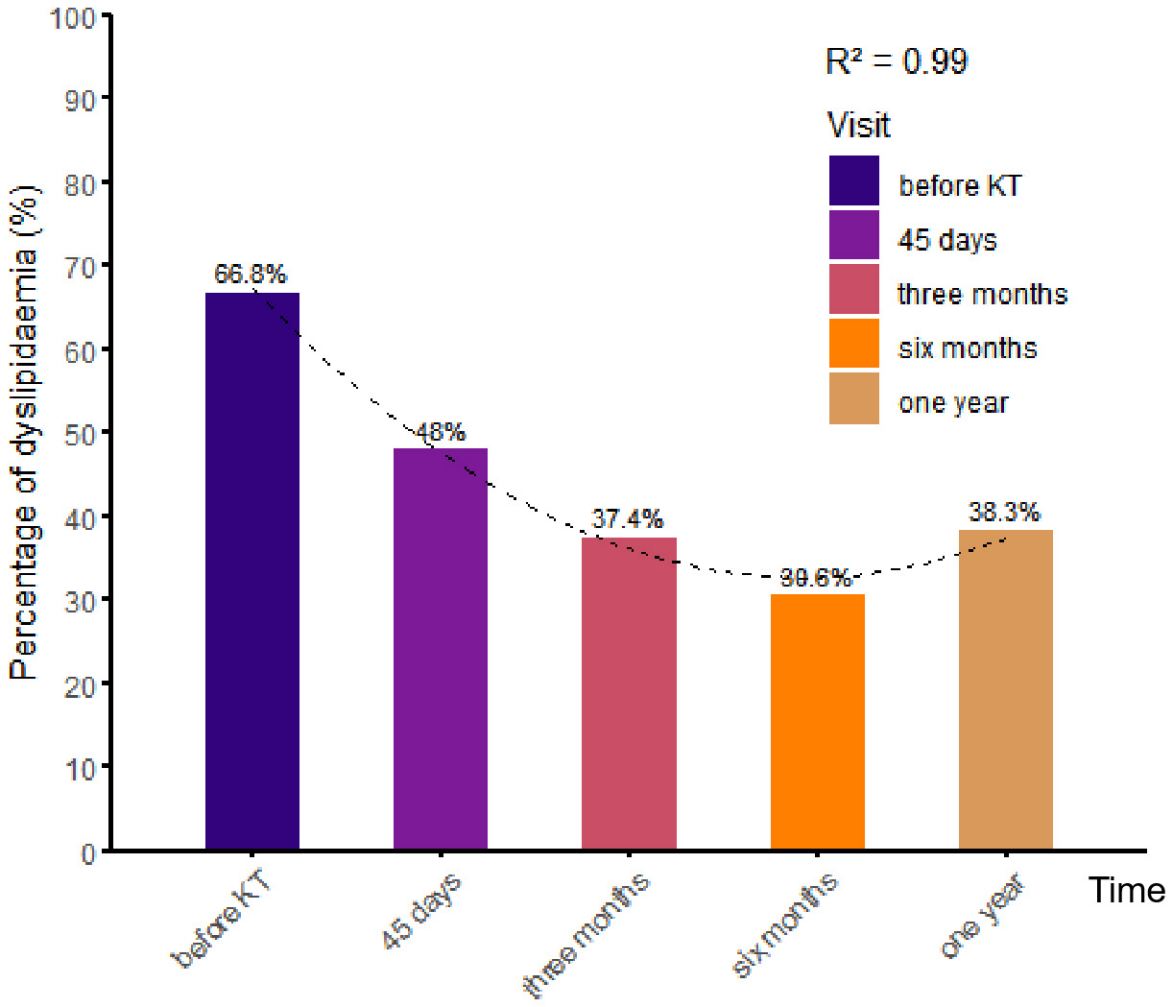
Different stages in 1-year incidence percentage of dyslipidemia. Visit 1: 45 days after surgery; visit 2: 3 months after surgery; visit 3: 6 months after surgery; visit 4: 1 year after surgery.

Our survey results indicate that approximately 66.8% of recipients had dyslipidemia prior to KT. However, the incidence of dyslipidemia after KT was 48.0%, 37.4%, 30.6%, and 38.3% at 45 days, 3 months, 6 months, and 1 year after discharge, respectively. Following KT, the prevalence of dyslipidemia declined compared to the pre-KT period. However, a subsequent increase in prevalence was observed (Figure 4).

**Figure 5:**
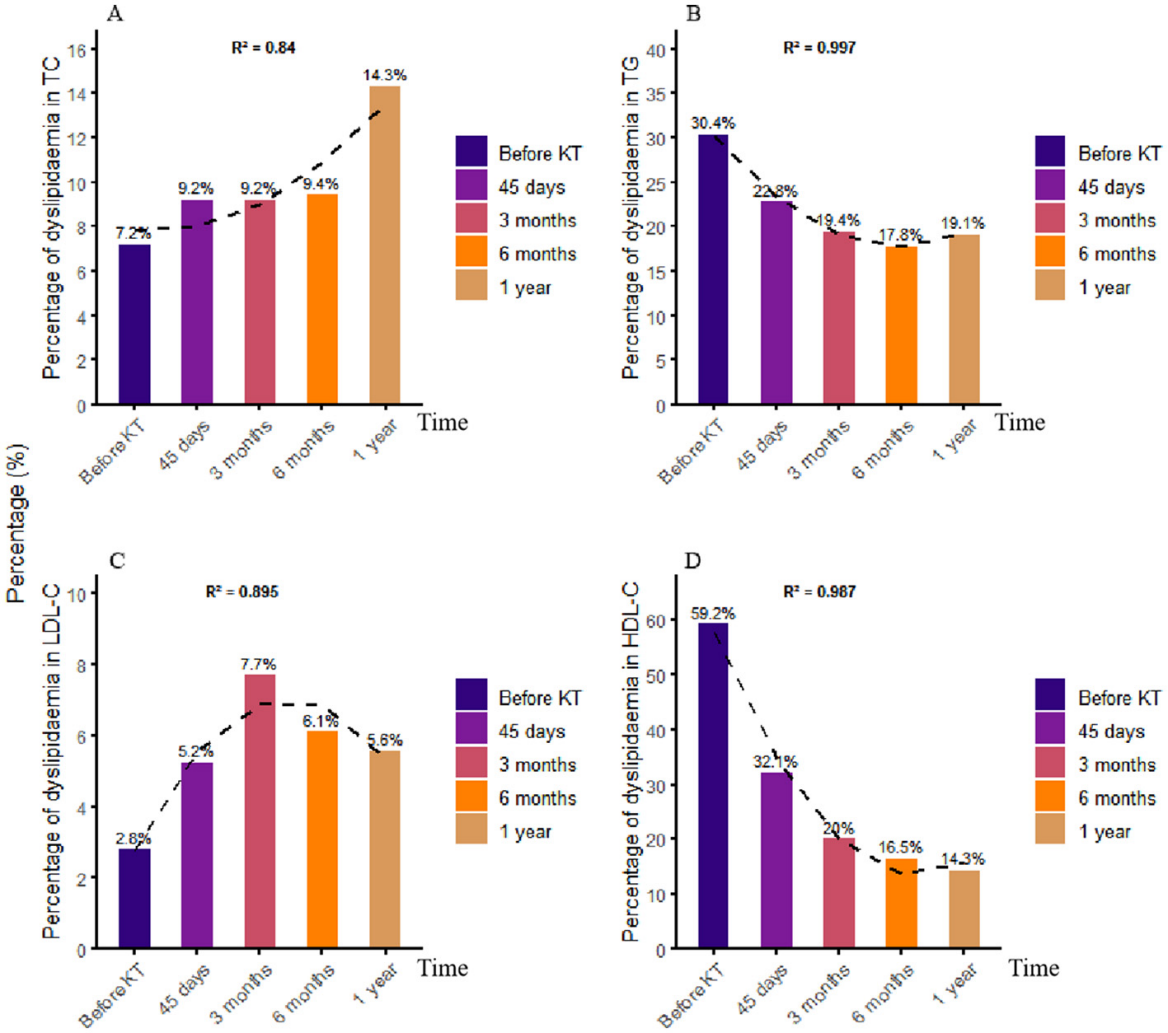
Incidence of excess lipid indices in kidney transplant recipients. This graph shows although the levels of the TG and HDL-C indices decreased after surgery, the increase in the TC and LDL-C indices cannot be ignored. **(A)** Percentage of values ≥6.2 mmol/l in TC. **(B)** Percentage of values ≥2.3 mmol/l in TG. **(C)** Percentage of values ≥4.1mmol/L in LDL-C. **(D)** Percentage of values < 1.0mmol/l in HDL-C. TG, triglyceride; TC, total cholesterol; HDL-C, high-density lipoprotein cholesterol; LDL-C, low-density lipoprotein cholesterol; visit 1: 45 days after surgery; visit 2: 3 months after surgery; visit 3: 6 months after surgery; visit 4: 1 year after surgery.

To identify the lipid component responsible for the observed lipid disorders, the incidence of these disorders was calculated for each lipid parameter: TC, TG, HDL-C, and LDL-C. The prevalence of dyslipidemia was primarily influenced by TG and HDL-C levels prior to surgery, while TC and TG levels were the primary determinants post-surgery. Additionally, the prevalence of TC and LDL-C abnormalities increased compared to the pre-operative status. However, the prevalence of TG and HDL-C disorders declined compared to the pre-operative period (Figure 5).

### 3.2 Effects of different body composition on dyslipidemia

To examine the relationship between variables such as BMI, SM, SAT, VAT, IMAT, and dyslipidemia prior to KT, participants were grouped based on body composition. The upper quartile of muscle or adipose tissue area was categorized as the high group, while the lower or middle quartile was categorized as the low group. The segmentation cutoff values are presented in Supplementary Table S2.

Additionally, participants were categorized into the 1-year group based on their laboratory lipid values, comprising 170 participants who met the criteria for dyslipidemia and 80 who did not (Table 2). No significant differences were observed between the dyslipidemia and non-dyslipidemia groups in terms of age, sex, smoking, drinking, family history of hypertension, or previous events. However, the median BMI in the dyslipidemia group was significantly higher than that in the non-dyslipidemia group (24.2 vs. 22.6 kg/m^2^, *P* = 0.006).

**Table 2:** Demographic characteristics of participants 1-year post-kidney transplantation surgery.

Variables	Dyslipidemia (IQR)/*n*, % *n* = 170	Non-dyslipidemia (IQR)/*n*, % *n* = 80	*P*-value
Demographic data			
Gender (man)	60%	72.35%	0.806
Age (years)	43 (36.00–52.75)	43 (33.75–52.25)	0.657
Height (m)	1.72 (1.65–1.78)	1.72 (1.64–1.75)	0.055
Weight (kg)	70 (60–80)	65 (55.25–76.25)	0.009
BMI (kg/m^2^)	24.22 (21.29–26.69)	22.61 (19.83–25.4)	0.006
Smoking (%)	17.06%	15%	0.547
Drinking (%)	7.06%	6.25%	1
Family history			
Hypertensive in the family history (%)	2.94%	1.25%	1
Diabetes in the family history (%)	3.53%	0%	< 0.001
Kidney disease in the family history (%)	1.76%	3.75%	1
Liver disease in the family history (%)	1.18%	1.25%	1
Previous events			
Previous cardiovascular events (%)	7.65%	8.75%	0.919
Past history of liver disease (%)	13.53%	6.25%	0.746
Mode of dialysis (%)	HD:79.41% PD:14.12% HD+PD:2.94% Without: 3.53%	HD:78.75% PD:12.5% HD+PD:3.75% Without: 5%	0.884
Duration of dialysis (month)	24 (12–48)	14 (9.75–37.50)	0.228
Hypertension			
Systolic pressure (mmHg) (mean ± SD)	154.98 ± 24.95	153.94 ± 20.62	0.745
Diastolic pressure (mmHg) (mean ± SD)	98.71 ± 14.88	97.95 ± 14.18	0.704
Immunosuppressive regimens			
Tacrolimus-based group (%)	100%	100%	-
Methylprednisolone (%)	31.18%	37.5%	< 0.001
Prednisone (%)	57.06%	43.75%	
Prednisone and methylprednisolone (%)	11.76%	18.75%	
Biochemical and clinical data			
Blood glucose (mmol/L)	5.57 (4.87–6.81)	5.68 (4.64–6.36)	0.298
HbA1c (%)	5.2 (5–5.6)	5.15 (4.9–5.53)	0.130
Albumin (g/L)	44.95 (41.34–47.55)	44.25 (41.65–47.35)	0.682
Hepatitis C (%)	1.18%	0%	-
Taking β receptor antagonists (%)	25.88%	16.25%	0.191
Morphomic variables			
SMA (cm^2^) (mean ± SD)	139.77 ± 33.07	129.16 ± 33.35	0.019
IMAT area (cm^2^)	3.95 (2.48–5.79)	2.86 (1.80–4.82)	0.005
VAT area (cm^2^)	119.55 (76.61–191.04)	67.65 (23.57–153.88)	< 0.001
SAT area (cm^2^)	139.12 (92.26–179.14)	108.5 (78.09–154.18)	0.003
SMI (cm^2^/m^2^) (mean ± SD)	47.02 ± 9.12	44.47 ± 9.92	0.047
IMATI (cm^2^/m^2^)	1.31 (0.84–1.97)	1.05 (0.62–1.58)	0.010
VATI (cm^2^/m^2^)	41.19 (25.76–66.88)	24.32 (8.18–52.93)	< 0.001
SATI (cm^2^/m^2^)	46.58 (32.47–58.97)	37.36 (29.07–52.27)	0.007

The aforementioned markers were gathered before kidney transplant surgeries. Blood glucose ^*^measurements included random blood sugar levels and fasting blood glucose levels.

BMI, body mass index; HD, hemodialysis; PD, peritoneal dialysis; HbA1c, hemoglobin A1c; TG, triglyceride; TC, total cholesterol; HDL-C, high-density lipoprotein cholesterol; LDL-C, low-density lipoprotein cholesterol; SMA, Skeletal muscle area; IMAT, Intermuscular adipose tissue; VAT, Visceral adipose tissue; SAT, Subcutaneous adipose tissue; IQR, interquartile range; n, number; I, index.

Body composition measures of VAT, IMAT, SAT, and SM were significantly different between the groups (*P* < 0.001; *P* = 0.005; *P* = 0.003; *P* = 0.019). Similarly, the indices of VATI, IMATI, SATI, and SMI were also significantly different between the groups (*P* < 0.001; *P* = 0.01; *P* = 0.007; *P* = 0.047). Both types of adipose tissue (VAT, IMAT, and SAT) showed higher levels in the dyslipidemia group compared to the non-dyslipidemia group. However, in the non-dyslipidemia group, the muscle tissue area was larger (139.8 vs. 129.2 cm^2^). Additionally, the presence of diabetes in the family history and the use of hormone medication were identified as statistically significant factors.

### 3.3 Visceral adipose tissue area is a predictor of lipid levels

Factors with a *P*-value less than 0.1 in the univariate analysis were selected for model construction. Due to multicollinearity between height, weight, BMI, and various body composition areas (VAT, IMAT, SAT, and SM) and indices (VATI, IMATI, SATI, and SMI), four sets of models were constructed by adjusting for these factors (Supplementary Table S3). The final iteration of Model 1 included a family history of diabetes, VAT area, and IMAT area. Model 2, in its final form, included a family history of diabetes and VATI. Model 3, in its final iteration, included height, family history of diabetes, VATI, and IMATI. Model 4, in its final form, included family history of diabetes, VAT area, and IMAT area.

**Figure 6:**
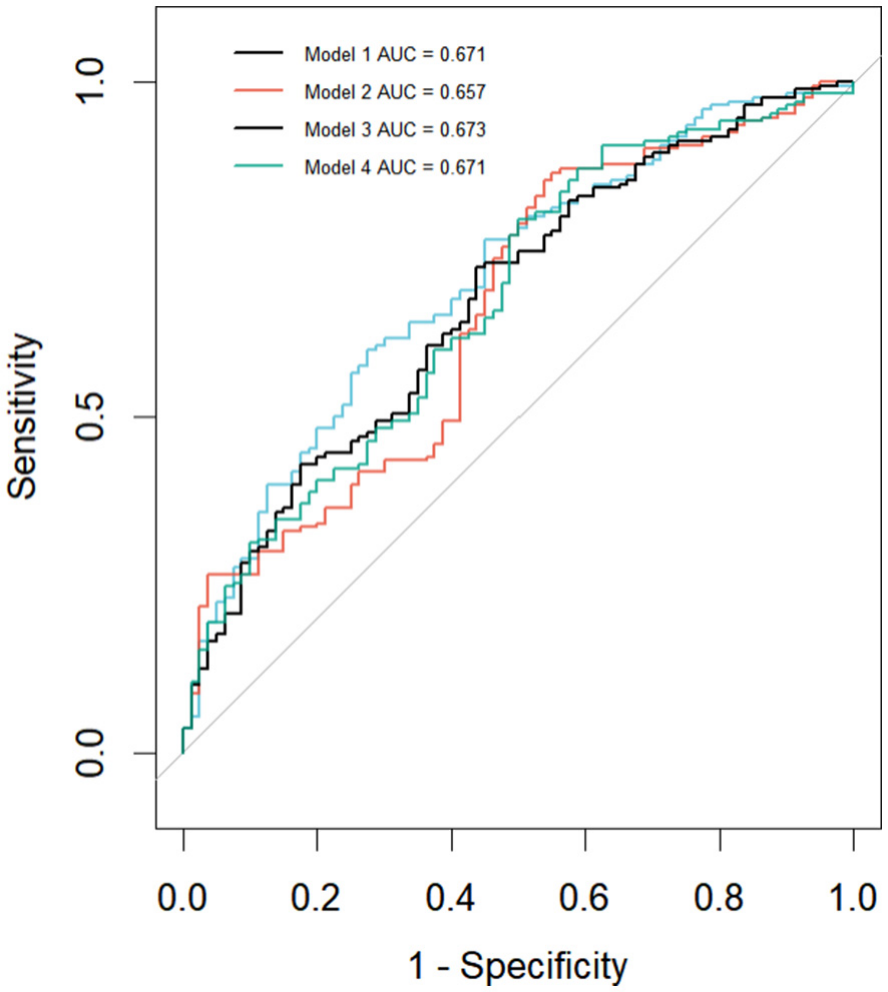
Receiver operating characteristic (ROC) curve of the dyslipidemia after kidney transplantation risk prediction model. This figure demonstrates that the predictive value of model 3 is better than that of models 1, 2, and 4.

The Omnibus test showed *P* < 0.001 for all four models, indicating that the models were collectively significant. The −2 log likelihood values were as follows: Model 3 < Model 4 < Model 1 < Model 2 (292.4 < 293.7 < 294 < 296; Table 3). Figure 6 demonstrates that the predictive ability of Model 3 is superior to that of the other three models (AUC 3 > AUC 1 = AUC 4 > AUC 2). However, only VAT was identified as an independent risk factor for dyslipidemia following KT in these models. The details of Model 3 are presented in Table 4.

**Table 3:** The risk prediction Modes for dyslipidemia after kidney transplantation.

Variables	Model 1		Model 2		Model 3		Model 4	
	OR (95%CI)	*P-*value	OR (95%CI)	*P-*value	OR (95%CI)	*P-*value	OR (95%CI)	*P-*value
Height (m)					15.018 (0.401–614.6)	0.146		
Weight (kg)								
BMI (kg/m^2^)								
Diabetes in the family history (%)	–	0.987	–	0.987	–	0.987	–	0.987
Methylprednisolone (%)								
Prednisone (%)								
Prednisone and methylprednisolone (%)								
SMA (cm^2^)								
IMAT area (cm^2^)	1.090 (0.973–1.238)	0.158					1.090 (0.973–1.238)	0.158
VAT area (cm^2^)	1.005 (1.001–1.009)	0.014					1.005 (1.001–1.009)	0.014
SAT area (cm^2^)								
SMI (cm^2^/m^2^)								
IMATI (cm^2^/m^2^)					1.324 (0.947–1.918)	0.119		
VATI (cm^2^/m^2^)			1.017 (1.007–1.028)	0.987	1.013 (1.002–1.025)	0.027		
SATI (cm^2^/m^2^)								
Intercept	–	0.986	–	0.987	–	0.983	–	0.986

BMI, body mass index; SMA, Skeletal muscle area; IMAT, Intermuscular adipose tissue; VAT, Visceral adipose tissue; SAT, Subcutaneous adipose tissue; IQR, interquartile range; n, number; I, index.

**Table 4:** Detailed parameters of Model 3.

Parameters	Regression coefficient	Standard error	Odds ratio	95% CI	*P*-value
Height (m)	2.709	1.864	15.018	0.401–614.6	0.146
Diabetes in the family history (%)	15.879	950.938	0.987	–	–
IMATI (cm^2^/m^2^)	0.280	0.180	1.324	0.947–1.918	0.119
VATI (cm^2^/m^2^)	0.013	0.006	1.013	1.002–1.025	0.027
Intercept	−20.679	950.944	0.00	–	0.983

IMAT, intermuscular adipose tissue; VAT, visceral adipose tissue; IQR, interquartile range; n, number; I, index.

To assess the accuracy of Model 3, the bootstrap method was employed, generating 1,000 samples through repeated sampling of the original dataset. The results showed that the model's ability to predict the occurrence of dyslipidemia after kidney transplantation was AUC = 0.696 (*P* = 0.039; 95% CI: 0.618–0.770). Additionally, the DCA curve in Supplementary Figure S1 demonstrates that Model 3 performs well when the threshold probability is between 37.5 and 87.5%.

To assess the detection capability of our study, we performed a sensitivity analysis. Based on the observed variance and sample size, the minimum detectable OR with 80% power was approximately 1.004 for VAT and 1.012 for VATI. The fact that our point estimates (VAT OR = 1.005; VATI OR = 1.013) marginally exceeded these values suggests that the study had sufficient sensitivity to identify these specific effects, albeit with power near the conventional threshold. This reinforces that the findings, while statistically significant, represent weak associations whose precise estimation and clinical relevance require verification in larger studies.

### 3.4 Long-term impacts of muscle and adipose tissue area on lipid levels

The results of the analysis comparing the high and low groups on LDL-C, HDL-C, TG, and TC levels during the follow-up period are presented in Table 5, indicating a statistically significant time effect between the two groups for TC, TG, HDL-C, and LDL-C. The data show that the levels of TC, TG, HDL-C, and LDL-C changed over time in both groups (Figure 6).

**Table 5:** High and low body composition groups of dyslipidemia generalized estimating equation results.

Variables		Time effect		Between-group effects		Crossover effect	
		*P-*value	*Wald X^2^*	*P*-value	*Wald X^2^*	*P-*value	*Wald X^2^*
VAT	TC (mmol/L)	< 0.001	50.436	0.213	1.548	0.346	4.468
	HDL -C (mmol/L)	< 0.001	223.400	0.001	11.775	0.857	1.324
	LDL -C (mmol/L)	< 0.001	104.948	0.027	4.877	0.065	8.855
	TG (mmol/L)	< 0.001	23.715	0.016	5.778	0.006	14.326
SAT	TC (mmol/L)	< 0.001	57.000	0.109	2.566	0.887	1.145
	HDL -C (mmol/L)	< 0.001	231.261	0.003	9.008	0.688	2.263
	LDL -C (mmol/L)	< 0.001	116.470	0.058	3.594	0.790	1.704
	TG (mmol/L)	< 0.001	27.305	0.001	10.351	0.002	16.636
IMAT	TC (mmol/L)	< 0.001	56.492	0.535	0.384	0.612	2.683
	HDL -C (mmol/L)	< 0.001	163.767	0.342	0.904	0.439	3.761
	LDL –C (mmol/L)	< 0.001	115.791	0.621	0.245	0.892	1.115
	TG (mmol/L)	< 0.001	21.308	0.120	2.411	0.172	6.390
SM	TC (mmol/L)	< 0.001	46.249	0.528	0.398	0.054	9.285
	HDL -C (mmol/L)	< 0.001	257.552	0.003	9.100	0.120	7.311
	LDL -C (mmol/L)	< 0.001	109.950	0.435	0.609	0.329	4.613
	TG (mmol/L)	< 0.001	20.991	0.185	1.761	0.131	7.086

The results of this generalized estimating equation demonstrate changes in TC, HDL-C, and LDL-C over time. It was found that LDL-C, HDL-C and TG levels were significantly different between the high and low VAT/SAT groups.

TC, total cholesterol; HDL-C, high-density lipoprotein cholesterol; LDL-C, low-density lipoprotein cholesterol; TG, triglyceride.

Notable discrepancies were observed in HDL-C, LDL-C, and TG levels between the high and low VAT groups (*P* = 0.001; *P* = 0.027; *P* = 0.016). HDL-C and TG levels differed between the high and low SAT groups (*P* = 0.003; *P* = 0.001; Figure 6, Supplementary Figure S2). HDL-C differed between the high and low SM groups (*P* = 0.003; Supplementary Figure S3). No statistically significant difference was observed in TC levels between the high and low VAT/SAT groups.

No statistically significant differences were observed in any of the lipids in the IMAT samples (Supplementary Figure S4). Similarly, no statistically significant differences were observed in TG, TC, and LDL-C levels between the high and low SM groups.

## 4 Discussion

Epidemiological analysis of dyslipidemia before and after KT revealed that its incidence exceeded 60% before KT, declined for 6 months post-discharge, and then showed an upward trend. This figure is somewhat lower than that reported in other studies (6), but it remains a significant concern. Further analysis revealed that the elevated incidence of pre-operative TG and HDL-C abnormalities contributed to the higher prevalence of pre-operative dyslipidemia. Post-operative dyslipidemia is primarily attributable to the emergence of elevated TG, TC, and HDL-C levels. The prevalence of dyslipidemia, indicated by elevated TC and LDL-C levels, increased post-operatively. Conversely, the prevalence of elevated TG and low HDL-C levels declined. Additionally, our analysis of KT recipients found that cardiovascular disease was the fourth leading cause of second hospitalization within a year, highlighting the importance of preventing cardiovascular disease before surgery. Our findings suggest that body composition factors such as BMI, VAT, SAT, IMAT, and SM levels prior to KT influence dyslipidemia. After adjusting for confounding factors, our results indicate that VAT may be a more robust predictor of risk. In other words, patients with a higher VAT mass are at greater risk of developing dyslipidemia. Further analysis showed that pre-transplant VAT was positively associated with TG and LDL-C, and inversely associated with HDL-C.

**Figure 7:**
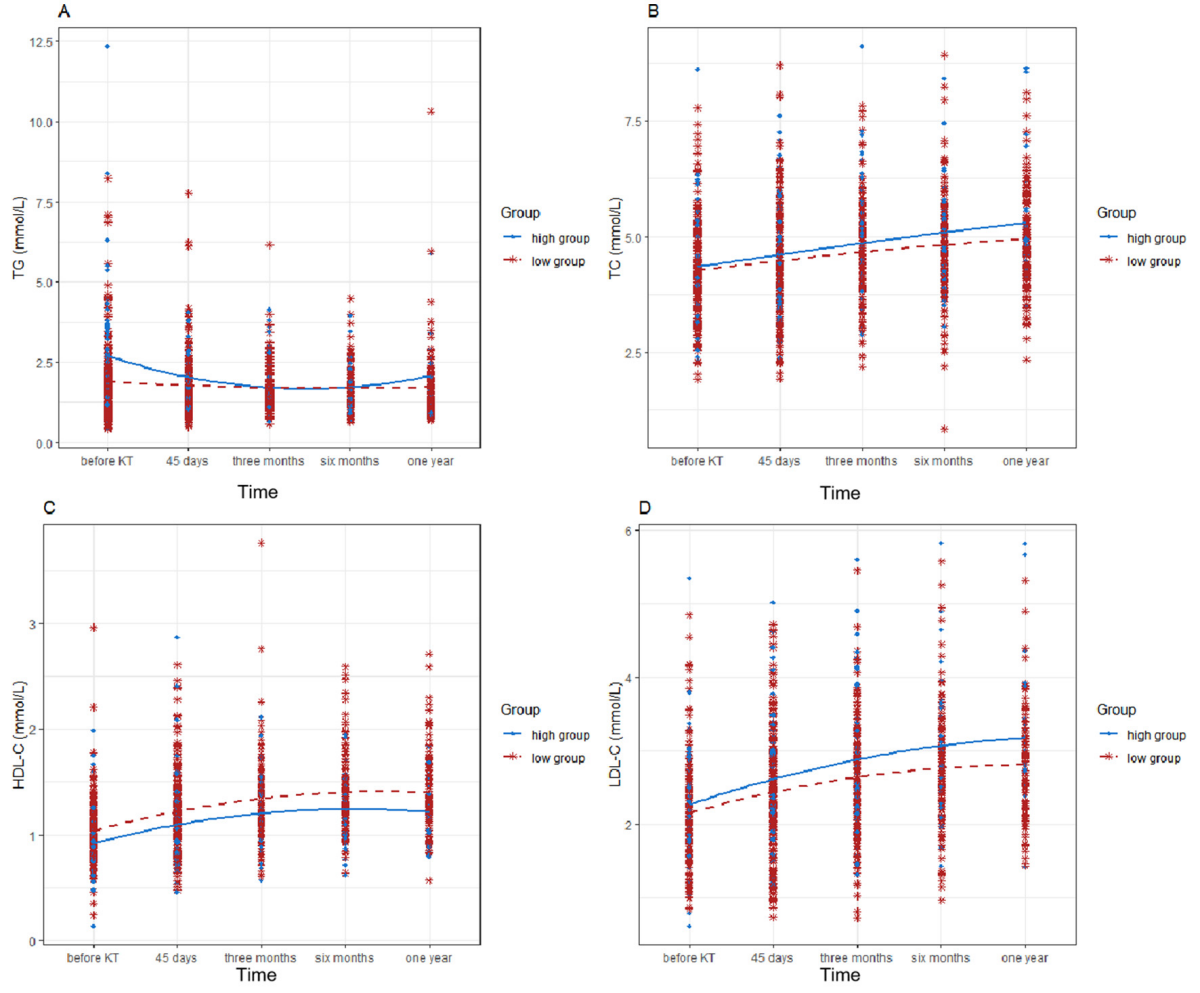
Diagram of the variations in blood lipid levels over time in High/Low VAT groups. This graph shows compared with low VAT, high VAT has different degrees of harm to TG, HDL-C, LDL-C. TG, triglyceride; TC, total cholesterol; HDL-C, high-density lipoprotein cholesterol; LDL-C, low-density lipoprotein cholesterol; visit 0: pre-operative imaging examination; visit 1: 45 days after surgery; visit 2: 3 months after surgery; visit 3: 6 months after surgery; visit 4: 1 year after surgery.

Our study demonstrated a gradual and sustained increase in TC and LDL-C levels over time (Figure 7), indicative of dyslipidemia. An investigation was then conducted to identify the underlying causes of this phenomenon. One theory is that immunosuppressive medications alter lipid metabolic pathways, leading to varying elevations in TG and LDL-C levels (22, 23). It was postulated that prolonged administration of immunosuppressive medications might contribute to this phenomenon. However, KT recipients must take immunosuppressants post-transplant, which inevitably affect blood lipids. One study reported an increased rate of obesity in KT recipients post-transplant (24). Consequently, another hypothesis suggests that obesity following KT is linked to elevated metabolic measures. Of the two factors, only obesity appears modifiable and may help prevent increases in TG and LDL-C levels. Although the increase in TG and LDL-C levels over 1 year is not significant enough to cause concern, the trend remains worrying. Prolonged dyslipidemia leads to ectopic fat deposition (lipotoxicity) in peripheral organs, such as the kidney, heart, and skeletal muscle, accelerating renal dysfunction and extrarenal complications, including renal anemia, heart failure, and sarcopenia (25). Thus, we anticipate that early intervention targeting VAT, a known risk factor for lipid disorders, will reduce CVD risk after KT.

BMI, a common measure of obesity, is strongly associated with dyslipidemia, a finding consistent with multiple studies (8, 26, 27). Our findings suggest that VAT is more strongly associated with dyslipidemia than BMI, supporting the findings of a 13-year cohort study (28). Additionally, a prediction model based on body composition was developed, showing greater accuracy. For example, VAT may be a more accurate predictor of type 2 diabetes risk and fistula development following pancreatoduodenectomy than BMI (29, 30). In our study, while both univariate BMI and body composition were correlated, body composition demonstrated superior performance after modeling.

The finding in this study that an increase in SM resulted in a significant rise in dyslipidemia is inconsistent with previous findings (31). However, a cross-sectional study involving 8,905 participants found that the body composition phenotype characterized by high muscle and high fat mass had the highest prevalence of dyslipidemia (32). Interestingly, this study suggests that, in individuals with normal fat content, an increase in muscle mass is associated only with a reduction in TC. However, when fat content is already high, TG significantly increase with muscle mass gain (*P* = 0.02). Furthermore, HDL-C significantly decreases with an increase in muscle mass (*P* < 0.05) (32). Our study aligns more closely with this explanation. The metabolic capacity of SM appears to favor fatty acid (FA) esterification and storage rather than oxidation (33). This suggests that the impact of SM on lipids may be influenced by adipose tissue. This study revealed that, compared to SM, VAT exhibits a stronger correlation with dyslipidemia following KT.

In contrast to VAT, which has growth potential, SAT has limited capacity for expansion. Excess triglycerides lead to lipid overflow, which accumulates in normal lean tissues such as the liver, heart (pericardium, epicardium, and myocardium), and SM—a process known as ectopic fat deposition (34). Research by Kim HS et al. on blood lipids revealed that ectopic fat accumulation in SM may be a significant risk factor for dyslipidemia and cardiometabolic diseases (35). The accumulation of adipose tissue between muscles leads to macrophage infiltration and the secretion of inflammatory factors (e.g., TNF-α, IL-6, IL-1β), which interfere with normal insulin signaling and lipid metabolism (36). Our study found a correlation between IMAT and dyslipidemia. However, this correlation is less significant than that of VAT. Previous studies using abdominal CT have shown that all intra- and per-organ fat accumulations correlate positively with VAT (37). In contrast, our study measured only fat accumulation between muscles, which was insignificant compared to VAT accumulation within and between organs. Thus, this study demonstrates that VAT, rather than IMAT, is more significant in establishing a model of dyslipidemia following KT.

The accumulation of VAT is a significant risk factor for CVD development, while dyslipidemia is a key precursor to CVD onset (37–39). A study in healthy individuals demonstrated an association between VAT and lipid laboratory values (10, 11). Another study in the KORA-MRI cohort reported that VAT plays a significant role in increasing dyslipidemia (40). However, no studies have yet investigated the relationship between pre-transplant VAT levels and the subsequent development of dyslipidemia following KT. Our study found that VAT is an independent marker of CVD risk and is associated with elevated LDL-C levels. These findings were confirmed by a cross-sectional study (41). We found that high pre-transplant VAT was associated with low HDL-C levels, consistent with a cross-sectional study of 538 participants over 3 years, which demonstrated an association between VAT and HDL-C (42). However, the study did not explore the relationship between VAT and long-term lipid levels in sufficient depth. Nonetheless, the association between elevated pre-transplant VAT and reduced HDL-C levels following KT was further substantiated by the cohort study design in this investigation. A study showed that HDL-C concentration was a significant predictor of future VAT accumulation over 5 years in Japanese Americans without diabetes (43). There appears to be an interaction between HDL-C and VAT. Thus, it is crucial to disrupt this cycle to prevent the development of CVD. Additionally, our findings indicated that elevated pre-transplant VAT was associated with elevated TG levels following KT in individuals with pre-existing high TG levels. One theory suggests that VAT accumulation, through vigorous lipolysis, exposes the liver to high concentrations of free fatty acids, leading to elevated TG levels (44). This study explores this theory.

This study has several limitations. Firstly, being a single-center retrospective design with a relatively limited sample size, the generalizability of its findings requires further validation in future multicenter, prospective studies. Although we compared baseline characteristics between patients who were followed up and those who were lost to follow-up and found no significant differences, the loss to follow-up of some patients may still introduce selection bias. Secondly, retrospective data collection inherently faces limitations in both the breadth and depth of variables captured. Several key factors known to be closely associated with lipid metabolism—such as detailed post-transplant dietary patterns, physical activity levels, weight dynamics, actual blood concentrations of immunosuppressive agents, and the use of lipid-lowering medications following dyslipidaemia onset—were not systematically recorded or analyzed. This may introduce residual confounding effects that complicate the interpretation of results. Thirdly, to serve early screening objectives, this study defined dyslipidaemia as at least one abnormal lipid parameter. While this composite endpoint enhanced screening sensitivity, it encompassed potentially heterogeneous phenotypes. Despite these limitations, to our knowledge, this represents the first systematic exploration of the association between body composition and post-operative dyslipidaemia in kidney transplant recipients. Moving forward, we shall conduct a multicenter prospective cohort study to systematically collect data on the aforementioned key exposure variables and clinical outcomes. This will incorporate more refined stratification of dyslipidaemia phenotypes to validate our findings and elucidate their underlying mechanisms.

In conclusion, our study found that VAT was a stronger predictor of dyslipidemia following KT over 1 year than other identified predictors. The longitudinal study of blood lipids revealed that VAT influences TG, LDL-C, and HDL-C levels. Although HDL-C levels increased over time, TC and LDL-C levels also rose, which may represent a disadvantage for KT recipients. Therefore, greater attention should be given to managing body composition in clinical practice, both before and after surgery. In the future, we will continue collecting data from KT recipients and follow them longitudinally to further investigate body composition and lipid disorders, as well as the consequences of dyslipidemia, including metabolic syndrome, cardiovascular disease, diabetes, and premature death, with the aim of developing a widely applicable predictive model.

## Data Availability

The datasets analysed in this study will be made available from the corresponding author upon reasonable request.
