# Supplementary material for: Association between pre-operative computed tomography-based adipose tissue quantification and post-transplant dyslipidemia in renal transplantation recipients
Source: Front Nutr. 2026 Mar 31;13:1729426. doi: 10.3389/fnut.2026.1729426 (PMC13076125; doi:10.3389/fnut.2026.1729426)
Supplement: Supplementary file 1 [file Data_Sheet_1.docx]

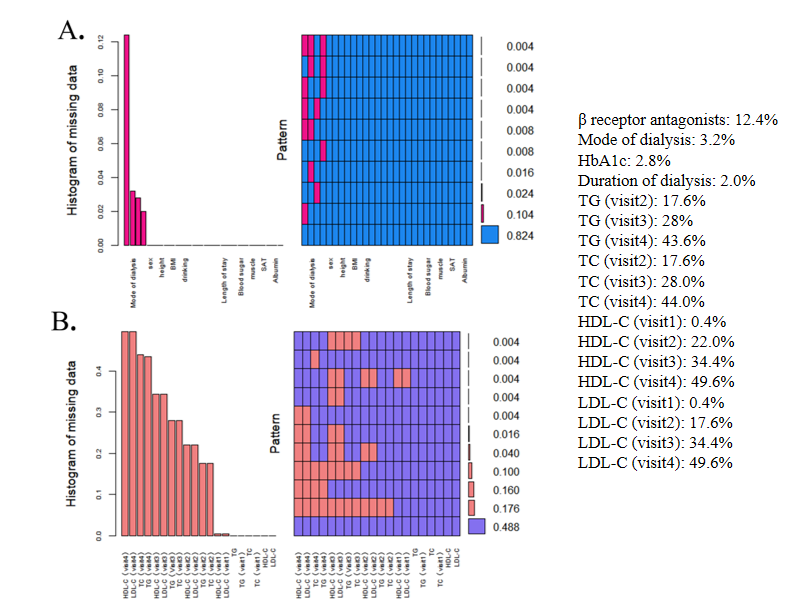


**Supplementary Figure 1. Summary of missing data.** A. missing variables for data collected preoperativelys; B. represent the absence of blood lipids values during follow-up periods. HbA1c, hemoglobin A1c; TG, triglyceride; TC, total cholesterol; HDL-C, high-density lipoprotein cholesterol; LDL-C, low-density lipoprotein cholesterol; visit 1: 45 days after surgery; visit 2: 3 months after surgery; visit 3: 6 months after surgery; visit 4: 1 year after surgery.


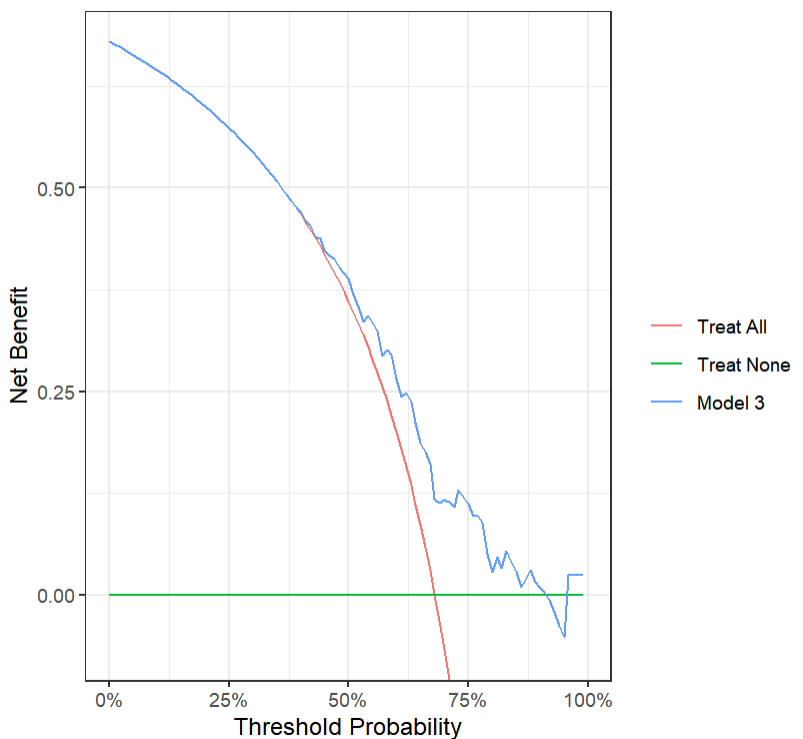


**Supplementary Figure 2.** Decision curve analysis of Model 3.


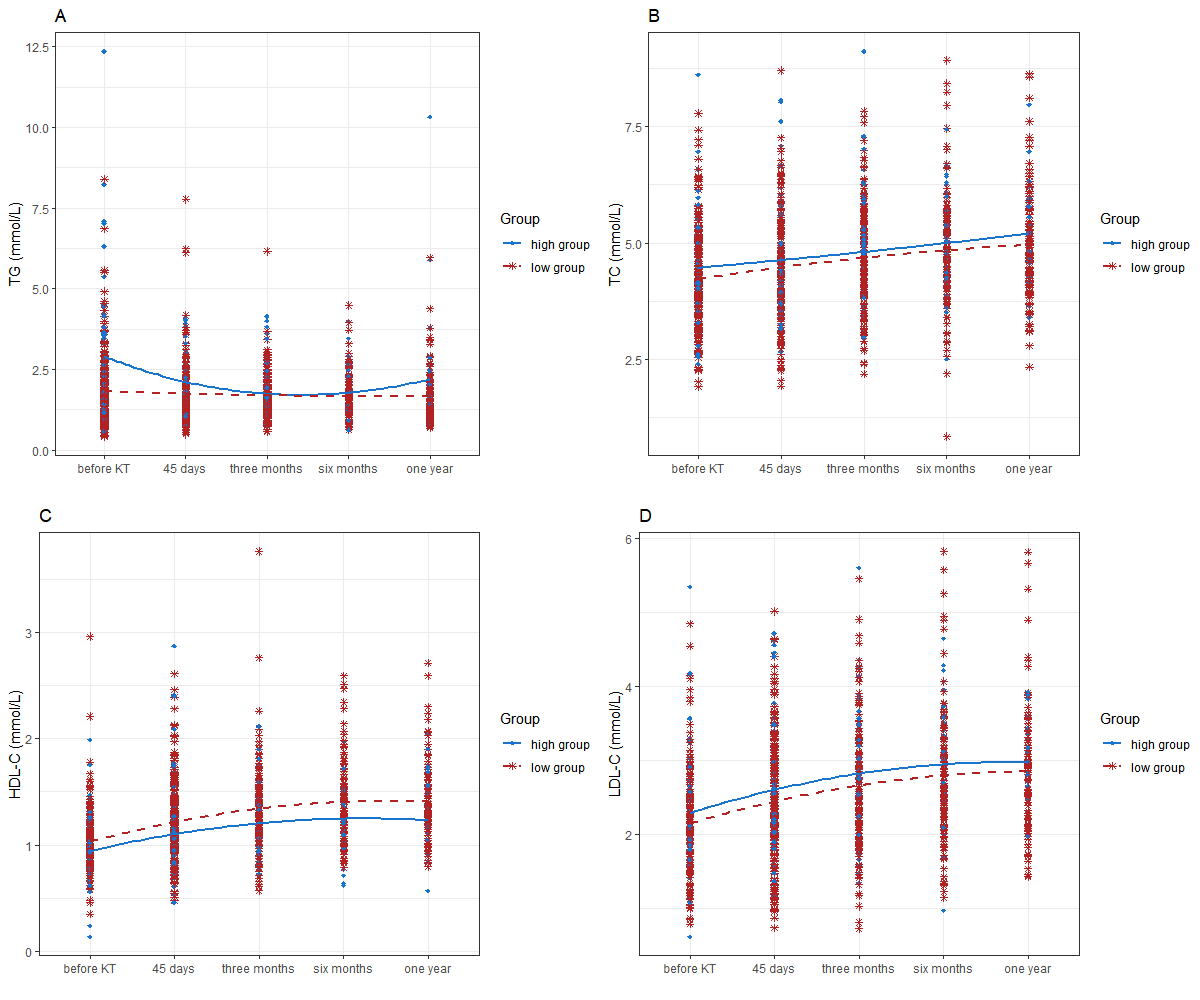


**Supplementary Figure 3.** Diagram of the variations in blood lipid levels over time in High/Low SAT groups. TG, triglyceride; TC, total cholesterol; HDL-C, high-density lipoprotein cholesterol; LDL-C, low-density lipoprotein cholesterol; KT, kidney transplantation.


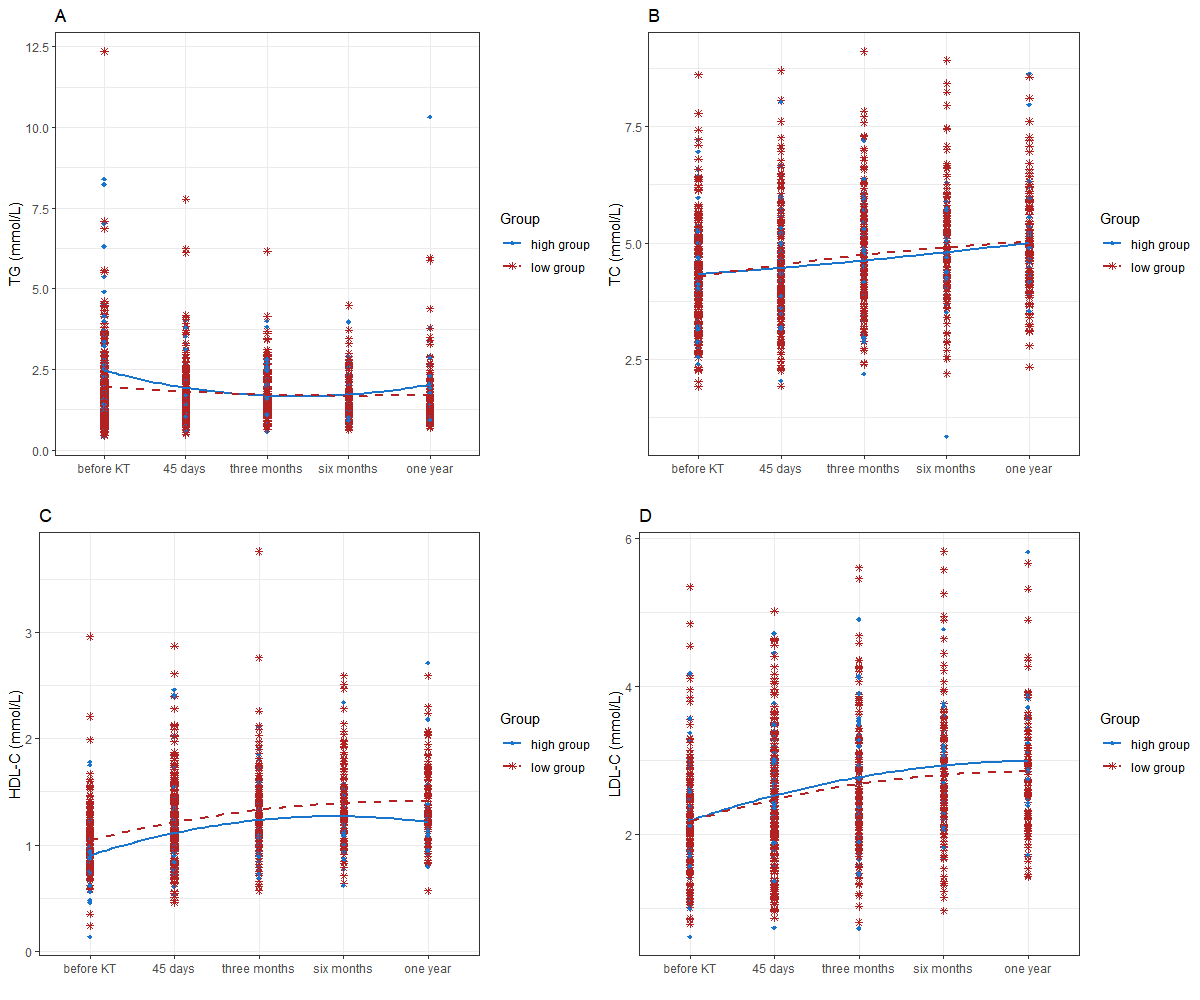


**Supplementary Figure 4. Diagram of the variations in blood lipid levels over time in High/Low SM groups.** TG, triglyceride; TC, total cholesterol; HDL-C, high-density lipoprotein cholesterol; LDL-C, low-density lipoprotein cholesterol; KT,kidney transplantation.

**
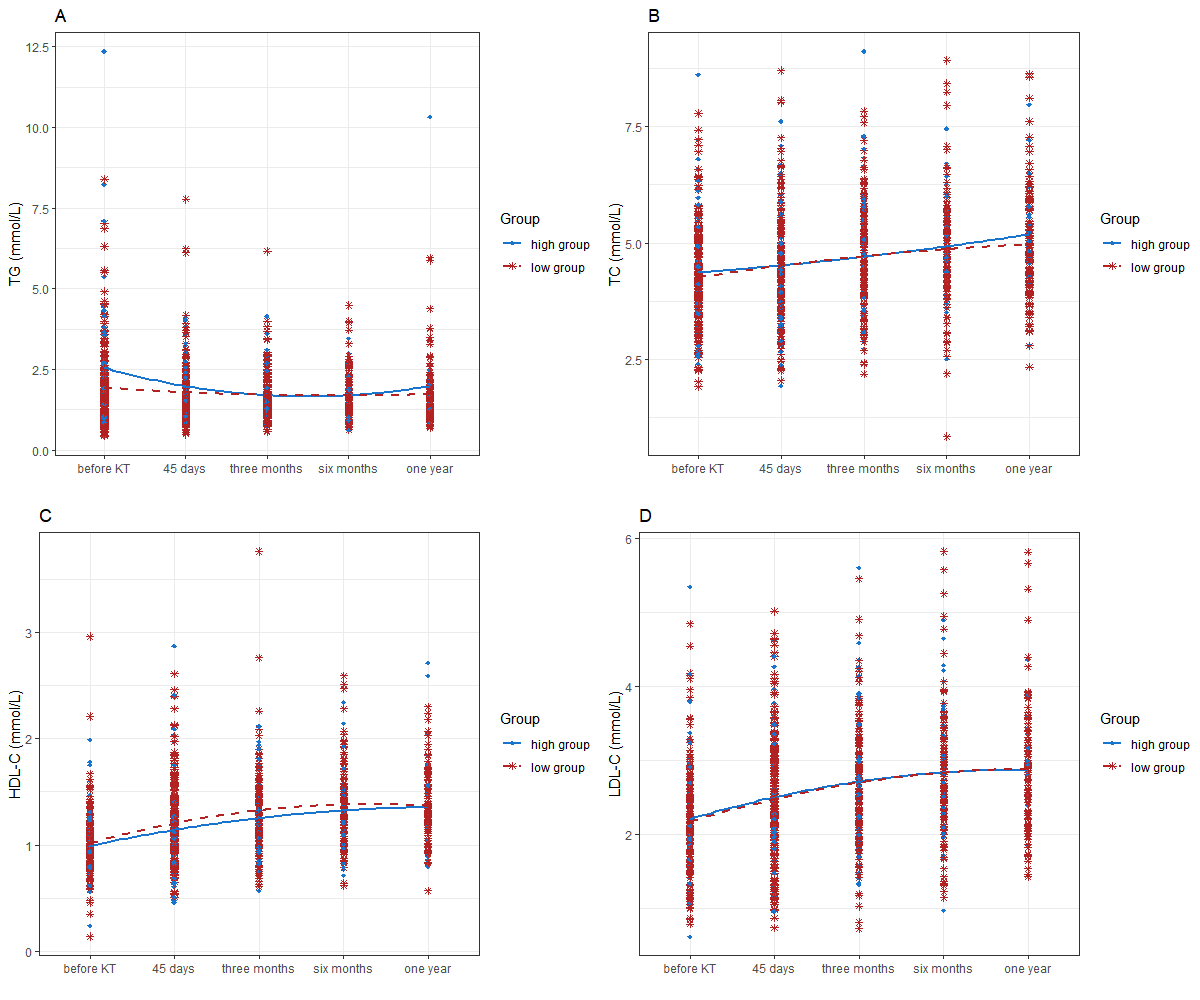
Supplementary Figure 5. Diagram of the variations in blood lipid levels over time in High/Low IMAT groups.** TG, triglyceride; TC, total cholesterol; HDL-C, high-density lipoprotein cholesterol; LDL-C, low-density lipoprotein cholesterol;KT,kidney transplantation.

**Supplementary Table 1. Demographic characteristics of participants who were lost to follow-up and those included in the study population**

| **variables** | **Lost to follow-up** | **Study population** | **P.value** |  |
| --- | --- | --- | --- | --- |
|  | **(IQR)/n, %** | **(IQR)/n, %** |  |  |
|  | **n = 62** | **n = 250** |  |  |
| **Demographic data** |  |  |  |  |
| Gender (man) | 75.81% | 68.40% | 0.255 | |
| Age (years) | 43.0(36.0-53.0) | 42.5(36.0-52.25) | 0.745 | |
| Height (m) | 1.72(1.65-1.78) | 1.72(1.65-1.77) | 0.898 | |
| Weight(kg) | 68.25(59.96-75.63) | 69.05(58.79-80.00) | 0.817 | |
| BMI (kg/m2) | 22.92(20.60-25.72) | 23.67(20.56-26.08) | 0.723 | |
| Smoking (%) | 22.58% | 16.40% | 0.253 | |
| Drinking (%) | 14.52% | 6.80% | 0.087 | |
| **Family history** |  |  |  | |
| Hypertensive in the family history (%) | 0% | 2.40% | 0.602 | |
| Diabetes in the family history (%) | 0% | 2.40% | 0.602 | |
| Kidney disease in the family history (%) | 1.64% | 2.40% | 1.000 | |
| Liver disease in the family history (%) | 3.28% | 1.20% | 0.254 | |
| **Previous events** |  |  |  | |
| Previous cardiovascular events (%) | 14.75% | 8.00% | 0.167 | |
| Past history of liver disease (%) | 6.56% | 11.20% | 0.404 | |
| Mode of dialysis (%) | HD:72.10% PD:16.40% | HD:79.80% PD:12.80% | 0.421 | |
|  | HD+PD:3.30% Without:8.2% | HD+PD:3.30% Without:4.1% |  |  |
| Duration of dialysis (month) | 14(5.75-48.00) | 24.0(12.0-48.00) | 0.118 | |

| **Biochemical and clinical data** |  |  |  |
| --- | --- | --- | --- |
| Blood glucose (mmol/L) | 5.50(4.83-6.63) | 5.57(4.81-6.59) | 0.917 |
| HbA1c (%) | 5.30(4.90-5.70) | 5.20(5.60-5.00) | 0.239 |
| Albumin (g/L) | 43.75(38.25-47.80) | 44.90(41.47-47.60) | 0.849 |
| Hepatitis C (%) | 0% | 0.8% | - |
| Taking β receptor antagonists (%) | 15.69% | 21.46% | 0.466 |

The aforementioned markers were gathered before kidney transplant surgeries. Blood glucose *measurements included random blood sugar levels and fasting blood glucose levels.

BMI, body mass index; HD, hemodialysis; PD, peritoneal dialysis; HbA1c, hemoglobin A1c; IQR, interquartile range; n, number; I, index .

| **variables** | **Quartile 1** | **Quartile 2** | **Quartile 3** |
| --- | --- | --- | --- |
| SMA (cm^2^) | 109.25 | 140.60 | 159.10 |
| IMAT area (cm^2^) | 2.251 | 3.680 | 5.388 |
| VAT area (cm^2^) | 49.45 | 112.38 | 180.26 |
| SAT area (cm^2^) | 87.11 | 128.30 | 168.30 |

**Supplementary Table 2. Quartile morphemic values according to the analysis of CT scans.**

This chart shows the quartile cut-off value of SMA, IMAT area, VAT area, and SAT area. Quartile 1 represents 25%, Quartile 2 represents 50%, Quartile 3 represents 75%.SMA, skeletal muscle area; IMAT, intermuscular adipose tissue; VAT, visceral adipose tissue; SAT, subcutaneous adipose tissue.

**Supplementary Table 3. Multicollinearity diagnostics for models.**

| **variables** | **VIF** | | | | | |
| --- | --- | --- | --- | --- | --- | --- |
|  |  | **Modle 1** | **Modle 2** | | **Modle 3** | **Modle 4** |
| Height (m) | 62.892 |  |  | 3.683 | | 2.899 |
| Weight(kg) | 695.810 |  |  | 7.088 | | 7.127 |
| BMI (kg/m^2^) | 419.817 | 4.133 | 3.962 |  | |  |
| Diabetes in the family history (%) | 1.000 | 1.000 | 1.000 | 1.000 | | 1.000 |
| Methylprednisolone (%) | 1.079 | 1.031 | 1.036 | 1.063 | | 1.064 |
| Prednisone (%) |  |  |  |  |  |  |
| Prednisone and methylprednisolone (%) |  |  |  |  |  |  |
| SMA (cm^2^) | 450.957 | 1.720 |  |  | | 2.776 |
| IMAT area (cm^2^) | 184.447 | 1.394 |  |  | | 1.400 |
| VAT area (cm^2^) | 264.706 | 2.415 |  |  | | 2.238 |
| SAT area (cm^2^) | 328.800 | 2.228 |  |  | | 2.478 |
| SMI(cm^2^/m^2^) | 308.282 |  | 1.819 | 1.813 | |  |
| IMATI (cm^2^/m^2^) | 182.685 |  | 1.414 | 1.421 | |  |
| VATI (cm^2^/m^2^) | 252.742 |  | 2.157 | 2.144 | |  |
| SATI(cm^2^/m^2^) | 324.620 |  | 2.298 | 2.458 | |  |

BMI, body mass index; SMA, Skeletal muscle area; IMAT, Intermuscular adipose tissue; VAT, Visceral adipose tissue; SAT, Subcutaneous adipose tissue; IQR, interquartile range; n, number; I, index.
